# Association between human papillomavirus DNA and temporal arteritis

**DOI:** 10.1186/1471-2474-13-132

**Published:** 2012-07-25

**Authors:** Amir Mohammadi, John D Pfeifer, James S Lewis

**Affiliations:** 1 Department of Pathology and Laboratory Medicine, University of Florida, College of Medicine, Jacksonville, Fl, USA; 2 Lauren V. Ackerman Laboratory of Surgical Pathology, Department of Pathology and Immunology, Division of Anatomic and Molecular Pathology, Department of Pathology and Immunology, Washington University School of Medicine, St. Louis, MO, USA; 3 Department of Otolaryngology – Head and Neck Surgery, Washington University School of Medicine, St. Louis, MO, USA

**Keywords:** Human papillomavirus, Giant cell arteritis, Polymerase chain reaction

## Abstract

**Background:**

To examine the relationship between human papillomavirus (HPV) and giant cell arteritis (GCA) of the temporal artery.

**Methods:**

The study group consisted of 22 cases of histologically positive/biopsy confirmed GCA. The control groups consisted of 21 histologically negative temporal artery biopsies and fifteen cases of vascular margins of nephrectomies. For detection of the presence of HPV, two methods were used: 1) polymerase chain reaction (PCR) with INNO-LiPA HPV Genotyping Extra, 2) Cervista™ HPV HR. All cases were from the files of the Barnes-Jewish Hospital and Washington University in St. Louis.

**Results:**

HPV DNA was detected by PCR and genotyping in 16 of 22 (73%) histologically positive cases of GCA and in only five of 21 (24%) histologically negative temporal artery biopsies. Among the vascular margin controls, only three of 15 (20%) were positive for HPV DNA. The second, independent method (Cervista^TM^) confirmed the aforesaid results with 100% concordance with the exception of three cases which had low genomic DNA for which it was not possible to perform the test. The differences in HPV positivity between the histologically positive and negative temporal artery biopsies and between the histologically positive temporal artery biopsies and controls were both statistically significant (p = 0.001 and 0.002, respectively).

**Conclusions:**

The results of our study revealed a statistically significant association between HPV positivity and biopsy confirmed temporal giant cell arteritis GCA (p = 0.001). Further studies are necessary to elucidate the pathophysiology underlying this association.

## Background

Giant cell arteritis is one of the most common causes of vasculitis involving the temporal artery. Other potential causes are Wegener granulomatosis, polyarteritis nodosa (PAN), and Buerger disease [1]. GCA generally affects individuals over 55 years of age (with a mean age at diagnosis of approximately 72 years) [2] with an annual incidence of approximately 18 per 100,000 in persons aged 50 years or older.

**Figure 1:**
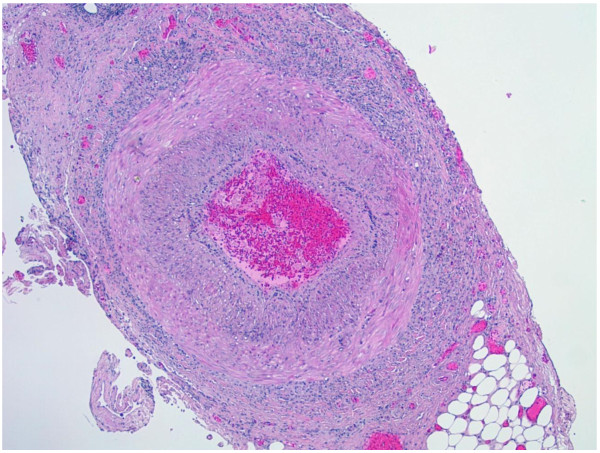
Biopsy of temporal artery showing a transmural mixed inflammatory cell infiltrate with intimal thickening, and fragmentation and distortion of the internal elastic lamina (H & E, original magnification x40).

**Figure 2:**
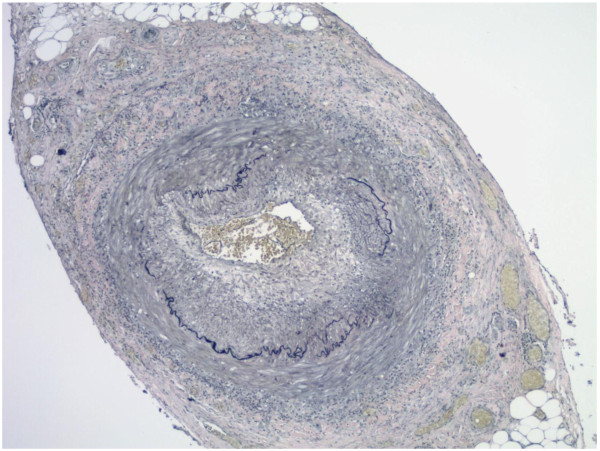
**Elastic stains on the same case of histologically positive temporal artery biopsy as shown in Figure**1**, showing the fragmentation, distortion and lack of continuity of the internal elastic lamina, a characteristic feature of temporal arteritis (Verhoeff-Van Gieson staining; original magnification, X40).**

Histologically, GCA shows transmural inflammation with mixed inflammatory cell infiltrate mostly consisting of lymphocytes, histiocytes, plasma cells, occasional neutrophils and rarely eosinophils (Figure 1). This causes destruction of the vessel's internal elastic lamina which is best demonstrated with elastic stains such as Verhoeff-Van Gieson (Figure 2) or Movat pentachrome. The presence of giant cells, next to the elastic lamina in particular, is the classic and pathognomonic feature of GCA. However, this is present in only about 50% of biopsy-proven cases.

Infectious agents have long been considered as a possible etiology of GCA. The concept is that GCA represents a chronic inflammatory response, triggered by an infectious agent, with subsequent inappropriate tissue response to injury. However, studies looking for organisms have had conflicting results. Some have demonstrated organism DNA, such as Herpes Simplex Virus, while the vast majority have failed to demonstrate an association. It is speculated that the inflammatory response may be triggered by an infectious agent. If a connection between GCA and HPV infection were to be established, the potential clinical implications are great, as GCA, with its possible vision loss [3-5], polymyalgia rheumatica and even eventually ischemic stroke [6-9], might potentially be prevented by vaccination or other strategies.

Human papillomavirus (HPV) is an increasingly common human pathogen in recent decades. It is a mucosotropic virus which is not thought of as spreading systemically. However, HPV genotype 16 has been found integrated into the genome of bacteria isolated from cervical cancer biopsies, and there is also published data showing HPV viral particles within peripheral nerves and small vascular endothelial cells adjacent to oral and cervical cancers as demonstrated by transmission electron microscopy [7,10]. In this study, we sought to determine if there is an association between GCA and HPV.

## Methods

With approval by the Washington University Human Research Protection Office (HRPO), we searched the Copath database of Barnes Jewish Hospital for temporal artery biopsy specimens from 1995 to 2008 and retrieved all specimens for which material was available. We identified approximately 60 cases, of which 43, (22 histologically positive and 21 histologically negative) had material available for review. There were five males and 17 females in the histologically positive GCA group with a mean age of 78.9 years. In the histologically negative group, there were four males and 17 females with a mean age of 67 years, and the control kidney vascular margin group had six males and nine females with a mean age of 38.2 years. There were no statistically significant difference in gender among the groups (p = 0.176). The clinical diagnosis of GCA was based on the criteria of the American College of Rheumatology [11]. However, for our study, we considered the gold standard to be histologic evidence of temporal arteritis. We also randomly selected 15 renal artery vascular resection margins from nephrectomy specimens in patients without any history of vasculitis for use as the negative controls.

Two methods were utilized to evaluate for HPV DNA. The first, INNO-LiPA HPV Genotyping Extra, was used to perform the testing in our research laboratory of the division of anatomic and molecular Pathology, Department of Pathology and Immunology, Washington University School of Medicine, St. Louis. All blocks were then submitted to an outside laboratory (CPA Laboratory, Louisville, KY) for independent preparation of genomic DNA and for testing employing the Cervista^TM^ HPV HR (Hologic, Madison, WI, USA) assay.

In both laboratories to avoid potential contamination, a maximum physical separation between the pre- and post-amplification steps was used. Separate pipettes and other lab materials were used as a part of good laboratory practice. The FFPE blocks were cut and processed under strict conditions to prevent DNA from being carried over from one case to the next during microtomy. Also a new blade was used for each case and the area was cleaned. Ice cubes used to cool blocks were discarded between cases.

With regards to the technical limitations of this study, it is well known that formalin fixation randomly fragments DNA in a duration-dependent manner, resulting in a partial degradation. The degree of fragmentation depends on the type and age of the sample and the conditions used for fixation. Due to this degradation, FFPE tissue is not suitable for amplification of large DNA segments. Nevertheless, PCR amplification of segments ranging up to 1300 base pair has been reported. On the other hand, incubation at an elevated temperature after proteinase K digestion partially removes formalin cross-linking of the DNA, improving yield as well as DNA performance in assays. Furthermore, in one of the techniques used in this study (INNO-LiPA HPV Genotyping), short-PCR-fragment (SPF 10) primers are employed, which amplify a 65 base pair segment of target DNA and this testing is considered to be one of the most sensitive PCR assays for the detection of HPV DNA.

### Washington University testing

#### DNA extraction

DNA was extracted using PureGene Kit (Gentra, http://www.Gentra.com) as per the manufacturer's instructions from 10 μm sections cut from the paraffin blocks. The concentration of the prepped DNA was measured spectrophotometrically using Nanodrop. Detailed procedure information is available at their web site, but briefly, we placed five 10 micron sections of tissue into a 1.5 mL microcentrifuge tube and added 1.0 mL of xylene, vortexed, and incubated for five minutes with constant gentle mixing. Then we centrifuged it for five minutes at 13,000-16,000 x g. In the fume hood, discarded by pipetting the xylene supernatant and left behind the visible pellet (tissue). We repeated this xylene wash twice. We then added 1.0 mL 100% ethanol, vortexed, and incubated five minutes with constant gentle mixing at room temperature, centrifuged at 13,000-16,000 x g for five minutes to pellet the tissue, and discarded the ethanol. We repeated these ethanol washes twice. Subsequently we added 1.0 mL 70% ethanol, gently mixed, and centrifuged at 13,000-16,000 x g for five minutes at 4°C, removed all residual ethanol and allowed tissue pellet to dry by centrifugation under vacuum for five minutes. For cell lysis, we added 300 μl Cell Lysis Solution (Gentra Puregene™ kit) and gently vortexed for 30 seconds. Then we added three μl Puregene Proteinase K (20 mg/ml), and mixed by inverting 25 times and incubated the lysate at 55°C for three hours to overnight. We inverted the tube periodically during the incubation. Then was added three μl RNase A Solution to the cell lysate, and mixed by inverting the tube 25 times and incubated at 37°C for 15 min to one hour. For Protein Precipitation we cooled quickly the sample to room temperature by placing on ice and added 100 μl Protein Precipitation Solution (Gentra Puregene™ kit) to the cell lysate. The volume should be 1/3 of the Cell Lysis Solution in the tube. Subsequently, it is vortexed vigorously at high speed for 20 seconds to mix the Protein Precipitation Solution uniformly with the cell lysate. Then, we centrifuged it at 13,000-16,000 x g for five minutes. The precipitated proteins formed a tight pellet. If the protein pellet was not tight, we vortexed vigorously for 20 seconds at high speed, and then incubated on ice for 5 min. We then centrifuged at 13,000–16,000 x *g* for three minutes. Using a pipette, we removed the supernatant containing the DNA (leaving behind the precipitated protein pellet) into a clean 1.5 mL microcentrifuge tube. We added two μl (for 400 μl supernatant) of a DNA carrier (glycogen; to final concentration of 50-150 mcg/μl) to aid recovery of small DNA quantities and then vortexed them. We then added 400 μl 100% of isopropanol. Subsequently, it was mixed by inverting gently ~50 times until the white threads of DNA formed a visible clump, and then it was centrifuged at 13,000-16,000 x g for 10 minutes and the supernatant was poured off. We added 500 μl of 70% ethanol and inverted the tube to wash the DNA pellet. We centrifuged at 13,000-16,000 x g for 5 minutes, and carefully poured off the ethanol and inverted and blotted the liquid from the tube on clean absorbent paper and allowed to air dry for 10-15 minutes. Finally, we added 50 μl DNA Hydration Solution and incubated at 65°C for one hour to dissolve the DNA. We incubated at room temperature overnight with gentle shaking. Samples could then be centrifuged briefly and transferred to a storage tube. The concentration of the DNA used for INNO-LiPA HPV Genotyping *Extra* testing in each case was 50 ng.

### INNO-LiPA HPV Genotyping testing

The INNO-LiPA HPV Genotyping *Extra* is based on the principle of reverse hybridization. Part of the L1 region of the human papillomavirus (HPV) genome is amplified using short-PCR-fragment assay (SPF10 primers), and the resulting biotinylated amplicons are then denatured and hybridized with specific oligonucleotide probes.

An additional primer pair for the amplification of the human HLA-DPB1 gene is added to monitor sample quality and extraction. The length of the HLA-DPB1 fragment is 280 base pairs. All probes are immobilized as parallel lines on membrane strips. After hybridization and stringent washing, streptavidin-conjugated alkaline phosphatase is added, which binds to any biotinylated hybrid previously formed.

Incubation with BCIP (5-Bromo-4-Chloro-3’-Indolyphosphate p-Toluidine Salt)/NBT (Nitro-Blue Tetrazolium Chloride) chromogen yields a purple precipitate, and the results are visually interpreted using the reference guide provided. An amplification kit (INNO-LiPA HPV Genotyping *Extra* Amp) is used for standardized preparation of biotinylated amplified material. This amplification kit is based on the polymerase chain reaction (PCR) using SPF10 primers.

**Figure 3:**
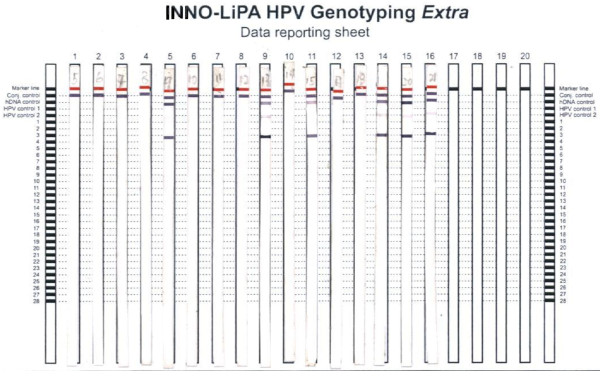
**INNO-LiPA typing on 16 patients. Strips # 5, 9, 11, 14, 15 and 16 are positive because they show clearly visible lines of hybridization.** The line patterns (corresponding to # 3 of the probe side bars) were compared to the interpretation chart supplied with the kit correspond with HPV type 16.

Amplification products are subsequently hybridized using a single typing strip on which 28 sequence-specific DNA probe lines and 4 control lines are fixed, which permits specific detection of 28 HPV genotypes, including all 18 high-risk genotypes, and 10 low-risk genotypes (HPV types 6, 11, 16, 18, 26, 31, 33, 35, 39, 40, 43, 44, 45, 51, 52, 53, 54, 56, 58, 59, 66, 68, 69, 70, 71, 73, 74, and 82) as described by the manufacturer (Figure 3).

### CPA Laboratory testing

#### DNA extraction

Slides were cut from the original blocks, and The QIAamp DNA FFPE Tissue kit (QIAGEN, http://www.qiagen.com) was used for purification of genomic DNA from formalin-fixed, paraffin-embedded tissues according to the manufacturer’s instructions with exception of incubation time. Overnight incubation in proteinase K for digestion of proteins/contaminants is not recommended by Qiagen but is something that CPA Laboratory has found useful to increase the nucleic acid elution.

Briefly, the QIAamp DNA FFPE Tissue procedure consisted of several steps including removal of paraffin from slides using xylene, subsequent specimen lysis under denaturing conditions with proteinase K, incubation at 90°C to reverse all formalin cross-linking, and DNA binding to the membrane for removal of contaminants. Residual contaminants were washed away and pure, concentrated DNA was eluted from the membrane.

### Cervista^TM^ HPV HR testing

Cervista^TM^ HPV HR [12] is a qualitative, diagnostic test for the detection of DNA from 14 high-risk HPV types (i.e., types 16, 18, 31, 33, 35, 39, 45, 51, 52, 56, 58, 59, 66, and 68). The Cervista^TM^ HPV HR test uses the Invader® chemistry which is a signal amplification technique for detection of specific nucleic acid sequences. In this method two types of isothermal reactions are used: a primary reaction that occurs on the targeted DNA sequence and a secondary reaction that produces a fluorescent signal. During the primary reaction, two types of sequence specific oligonucleotides (i.e. a probe oligonucleotide and an Invader® oligonucleotide) bind to the target DNA recognition site. When those sequence specific oligonucleotides overlap by at least one base pair on the target sequence, an invasive structure forms that acts as a substrate for the Cleavase® enzyme. The enzyme cleaves the 5’ portion (flap) of the probe at the position of the overlap.

The probes are present in large molar excess and cycle rapidly on and off the target sequence generating many cleaved 5’ flaps per target sequence. The cleaved flaps then bind to a universal hairpin fluorescence resonance energy transfer (FRET) oligonucleotide creating another invasive structure that the Cleavase® enzyme recognizes as a substrate. The enzyme cleaves the FRET oligonucleotides between the fluorophore and quencher molecule and produces a fluorescence signal as the cleaved flaps cycle on and off. For each copy of target, the combined primary and secondary reactions result in 10^6^– 10^7^ fold signal amplification per hour. The reagents for this test are provided as three oligonucleotide master mixtures, which identify the 14 types of HPV arranged according to phylogenetic relatedness. Master mixture 1 identifies the positivity of genotypes 51, 56 and 66(MM1: HPV 51, 56 and 66). Master mixture 2 (MM2) shows the positivity of genotypes HPV 18, 39, 45, 59 and 68 and master mixture 3 (MM3) reveals the positivity of HPV genotypes 16, 31, 33, 35, 52 and 58. By design, the released 5'-flaps bind only to their respective FRET oligonucleotides to generate target-specific signal. A positive result indicates that at least one of the 14 high-risk types is present in the DNA sample. For each case 150 to 200 ng of DNA prepped from the FFPE tissues at CPA Labs were utilized for the Cervista HPV testing. The sensitivity of this test is set at 5000 copies of HPV DNA. The Cervista assay has an internal control to verify if the assay worked. If the internal control does not pass, it suggests either the sample is degraded or extraction failed or the PCR did not work.

### Statistics

Statistical analyses were performed using the Chi-Square, *T*-test and Fisher's exact tests to evaluate the correlation between the presence of HPV DNA and other variables. *P* values < 0.05 were considered significant. All statistical analyses were performed using the SAS v.9.1 system software (SAS Institute Inc., Cary, NC).

## Results

Using the INNO-LiPA HPV Genotyping *Extra,* 16 of the 22 (17 female and 5 male) biopsy-confirmed GCA cases were positive for HPV. Twelve out of the 16 (75%) HPV-positive GCA-positive patients were female. Among these 16 positive cases, 5 had a HPV 6 alone (31%), 3 cases had HPV 16 alone (19%), 7 cases had both types 6 and 16 (44%), and only one case was extensively "multi-genotype" showing types 6, 16, 31, 33, and 40 (6%) (Testing results are presented in Tables 1 and 2). In all cases where HPV was detected, type 6, type 16, or both were present (100%). The cases were also tested by the Cervista^TM^ HPV HR assay for confirmation, which only evaluates for high risk HPV types (not for HPVs 6 and 11). Two cases which were HPV type 6 by INNO-LiPA (cases # 4 and 6) had low genomic DNA when prepped for the Cervista^TM^ HPV HR testing, so could not be evaluated by this method. All positive cases of the Cervista^TM^ HPV HR were from the Master Mix 3 which includes the HPV genotypes 16, 31, 33, 35, 52 and 58. As expected, all cases for which genotype 6 was present in the INNO-LiPA method were negative with the Cervista method since low risk types are not evaluated in that assay. All negative cases with INNO-LiPA HPV Genotyping *Extra* were negative also with Cervista^TM^ HPV HR testing. As such, there was 100% agreement between the two testing methods on the histologically positive temporal arteritis cases.

**Table 1:** **Results of the HPV testing by both different methods**.

**Histologically Positive GCA cases**	**INNO LiPA Results**	**Cervista Results**	**Histologically Negative GCA cases**	**INNO LiPA Results**	**Cervista Results**	**Kidney Vascular margin**	**INNO LiPA Results**	**Cervista Results**
**1**	**Neg**	**Neg**	**1**	**Neg**	**Neg**	**1**	**Neg**	**Neg**
**2**	**6**	**Neg**	**2**	**Neg**	**LGD**	**2**	**Neg**	**Neg**
**3**	**Neg**	**Neg**	**3**	**Neg**	**Neg**	**3**	**Neg**	**Neg**
**4**	**6**	**LGD**	**4**	**6**	**Neg**	**4**	**Neg**	**Neg**
**5**	**6, 16, 31, 33, 40**	**pos MM3**	**5**	**Neg**	**Neg**	**5**	**Neg**	**Neg**
**6**	**6**	**LGD**	**6**	**52**	**pos MM3**	**6**	**Neg**	**Neg**
**7**	**6**	**Neg**	**7**	**Neg**	**Neg**	**7**	**Neg**	**Neg**
**8**	**6**	**Neg**	**8**	**Neg**	**Neg**	**8**	**6**	**Neg**
**9**	**Neg**	**Neg**	**9**	**Neg**	**Neg**	**9**	**16**	**pos MM3**
**10**	**6, 16**	**pos MM3**	**10**	**Neg**	**Neg**	**10**	**Neg**	**Neg**
**11**	**16**	**pos MM3**	**11**	**Neg**	**Neg**	**11**	**16**	**pos MM3**
**12**	**6, 16**	**pos MM3**	**12**	**Neg**	**Neg**	**12**	**Neg**	**Neg**
**13**	**16**	**pos MM3**	**13**	**Neg**	**Neg**	**13**	**Neg**	**Neg**
**14**	**6, 16**	**pos MM3**	**14**	**Neg**	**Neg**	**14**	**Neg**	**Neg**
**15**	**Neg**	**Neg**	**15**	**Neg**	**Neg**	**15**	**Neg**	**Neg**
**16**	**Neg**	**Neg**	**16**	**16**	**pos MM3**			
**17**	**Neg**	**Neg**	**17**	**Neg**	**Neg**			
**18**	**6, 16**	**pos MM3**	**18**	**16**	**pos MM3**			
**19**	**6, 16**	**pos MM3**	**19**	**Neg**	**Neg**			
**20**	**6, 16**	**pos MM3**	**20**	**16**	**pos MM3**			
**21**	**16**	**pos MM3**	**21**	**Neg**	**Neg**			
**22**	**6, 16**	**pos MM3**						

*LGD*: Low Genomic DNA.

*POS MM3*: Positive in Master Mix 3.

*HPV* = Human papillomavirus.

MM1: HPV types 51, 56 and 66.

MM2: HPV types 18, 39, 45, 59 and 68.

MM3: HPV types 16, 31, 33, 35, 52 and 58.

**Table 2:** Correlation between the three study groups for HPV test results

	Biopsy Positive GCA*	**Biopsy Negative GCA**	**All Temporal Artery Biopsies**	Renal Artery Controls**
HPV Positive	16 (73%)	5 (24%)	21(49%)	3
HPV Negative	6 (27%)	16 (76%)	22 (51%)	12
Total	22	21	43	15

*Differences between biopsy positive and biopsy negative cases and between biopsy positive and control cases were statistically significant ((p = 0.001 and 0.002, respectively).

**Difference between biopsy negative and control cases was not statistically significant (p = 0.79).

*GCA* = giant cell arteritis; *HPV* = human papillomavirus.

Among the 21 cases of GCA histologically negative biopsies, 5 were positive for HPV by INNO-LiPA HPV Genotyping *Extra*, of which 3 (60%) were genotype 16, one (20%) was genotype 6 and one (20%) was genotype 52. Cervista^TM^ HPV HR testing revealed positivity of Master Mix 3 for all 4 cases which were positive by INNO-LiPA HPV Genotyping *Extra* for high risk HPV. Case number 2 which was negative with INNO-LiPA was of low genomic DNA with Cervista method. As such, there was 100% agreement between the two testing methods on the histologically negative temporal artery biopsy cases.

Among the 15 nephrectomy vascular resection margins (controls), 3 were positive for HPV by INNO-LiPA HPV Genotyping *Extra*. Two cases showed genotype 16 (1 case of Wilms' tumor and 1 of acute and chronic pyelonephritis), and one genotype 6 (a case of papillary urothelial carcinoma). Cervista^TM^ HPV HR testing revealed positivity of Master Mix 3 for both cases which were positive for high risk HPV by INNO-LiPA HPV Genotyping *Extra.* As such, there was 100% agreement between the two testing methods on the 15 vascular margin control cases.

The association between HPV positivity and histologically confirmed GCA was statistically significant (p = 0.0013). There was also a statistically significant association between HPV positivity and Caucasian race (p = 0.0339). No other associations were statistically significant.

In multivariate analysis (Table 3), subjects with histologically confirmed GCA had a 56-fold higher likelihood of having HPV positivity adjusted by gender, age and race (OR, point estimate 56.01; 95% CI 3.5-895.68).

**Table 3:** Odds Ratios for variable and presence of histologically positive temporal artery biopsies

**Variable**	**Point Estimate**	**95% Wald Confidence Interval**	
**Gender F*vs.*M**	**2.762**	**0.329**	**23.222**
**Race: African American*vs.*Caucasian**	**0.113**	**0.006**	**2.199**
**HPV**	**56.014**	**3.503**	**895.681**
**Age**	**1.196**	**1.053**	**1.358**

*HPV* = human papillomavirus.

## Discussion

Giant cell (temporal) arteritis is a chronic vasculitis involving medium and large size arteries that typically affects individuals older than 50 years of age.[1,3,4] Although it usually affects the superficial temporal arteries, it can also affect the aorta, carotid, subclavian, vertebral, and iliac arteries. The classical picture of granulomatous inflammation with multinucleated giant cells is observed in approximately 50% of the patients.

Histologically, the disease progresses from minimal involvement of vessels, with only collections of lymphocytes confined to the internal or external elastic lamina or adventitia to a panarteritis with segmental areas of necrosis of the arterial wall and extensive destruction of the elastic laminae, a feature that can be clearly demonstrated with special stains for elastic fibers (Figures 1 and 2).

The etiology of the inflammatory reaction in the GCA has not been identified. However, it has been demonstrated that there is a restricted clonal expansion of tissue-infiltrating T cells in these lesions, which suggests that they are reacting to a specific antigen located within the affected arterial wall which is thus eliciting the disease [13]. The most recent hypothesis regarding the etiology of GCA contends that a response to endothelial injury (maybe due to infection) leads to an inappropriate activation of T-cell-mediated immunity [14,15]. The subsequent release of inflammatory mediators within the arterial vessel wall can attract macrophages which then become multinucleated giant cells, creating the characteristic histology of this disease and also leading to an oligoclonal expansion of T-cells directed against antigens within or near the elastic lamina. This cascade of events in due course results in vessel wall damage, intimal hyperplasia, and eventual stenotic occlusion.

Infectious agents have been considered in the past as the etiology of GCA. Elling et al., by indirect serological evidence, found high incidence of GCA within a population associated with *Chlamydia pneumoniae*, *Mycoplasma pneumoniae *, and Parvovirus B19 [16]. Published data from Wagner et al. [15] and Powers et al. [17] reported a relationship between GCA, *C. pneumonia* and herpes simplex virus (HSV). However, further studies were unable to detect the presence of these infectious agents. Cooper et al. [14] and Cankovic and Zarbo [18], could not confirm the association between GCA and *C. pneumonia*, HSV, varicella zoster virus (VZV), Epstein–Barr virus (EBV), or human herpesvirus 7 (HHV7). In addition, Rodriguez-Pla et al. [19], Helweg-Larsen [20] and other authors [21-23] could not confirm the presence of herpes viruses, varicella zoster virus, parvovirus, or *C. pneumoniae* in temporal artery GCA. Due to this conflicting and contradictory data, no conclusive link has, to date, been demonstrated between a GCA and a specific infectious agent.

The role of human papillomavirus (HPV) in the development of cervical, head and neck, anal and skin cancers is well known. Molecular and epidemiological studies have shown that persistent HPV infection is the most important risk factor for cervical cancer [24,25]. High risk HPV also has an important role in anogenital and oropharyngeal squamous cell carcinoma [26]. They are also implicated with skin cancer in individuals with epidermodysplasia verruciformis [27] and can induce a variety of proliferative lesions, such as warts and laryngeal papillomas. There are very few associations between HPV and non-neoplastic diseases.

Interestingly, Ma et al. [10] reported the presence of HPV type 16 in the genome of bacterial strains (*Enterococcus *, *Staphylococcus *, *Bacillus* and *Corynebacterium*) and demonstrated the HPV viral particles by transmission electron microscopy in those bacteria. In addition, HPV type 16 has been detected in peripheral nerves, and vascular endothelial cells [7]. Another possible pathway is for HPV to disseminate systemically. This has long been debated [28]. The role of viremia in the pathogenesis of HPV-related diseases is still unclear, although HPV DNA has been detected in peripheral blood in some studies, though in varying amounts [29-32].

In this study, we found that HPV DNA, of both high and low risk types, is present in formalin-fixed, paraffin-embedded temporal artery biopsy specimens, and in statistically significantly higher numbers in the arteritis specimens compared to histologically-negative temporal artery biopsies and nephrectomy vascular margin controls. The validity of these results was confirmed with exactly matching results by an outside laboratory that independently prepped DNA from the paraffin blocks and utilized a completely different detection method. Our results are admittedly surprising and bring many questions about what HPV's role, if any, would be in temporal arteritis. HPV has been associated with tumorigenesis, and many studies have investigated HPV-related modification of the immune system to establish infection. However, we are not aware of any literature citing HPV as an inciting agent for inflammatory diseases. Our findings do support the notion that HPV can disseminate systemically, but do not, by themselves, tell us anything specific about the pathophysiological relationship between the virus and temporal arteritis, if there even is one. Further studies are necessary to address this.

It is important to mention that a negative temporal artery biopsy does not rule out the diagnosis of temporal artery GCA, since the changes can be patchy with skip areas of uninvolved artery. Although serial sectioning of the temporal artery biopsy is recommended, not all cases will be diagnosed histologically [33]. Actually, after review of the medical records of five of our biopsy-negative HPV-positive cases, in one case, the patient showed dramatic improvement after treatment with steroids (possible false negative case which was not included as a HPV-positive case in our statistical analysis).

It is also worth noting that of the 16 histologically-proven GCA cases that had HPV, 11 were high risk genotype 16. Only five cases were positive for low risk HPV genotype 6. Although, the importance of distinguishing low risk from high risk HPV genotypes has been established in the pathogenesis of neoplasms, what its significance might be in inflammatory disorders such as GCA is currently unknown.

## Conclusion

In summary, we have identified HPV DNA in the majority of histologically-proven giant cell (temporal) arteritis specimens and validated these results by a completely independent outside laboratory assay. The association raises questions regarding the biology of HPV infections, when dissemination occurs, and what this dissemination means clinically. Such studies are required to understand the significance of the association in our series.

## Competing interests

The authors declare that they have no competing interests.

## Authors’ contributions

AM conceived the study, carried out the molecular testing, analyzed the data, and drafted the manuscript. JDP and JSL, designed the study, collected data, helped interpret findings and critically revised the manuscript. All authors read and approved the final manuscript.

## Pre-publication history

The pre-publication history for this paper can be accessed here:

http://www.biomedcentral.com/1471-2474/13/132/prepub

## References

[B1] Burke A Virmani R Mills SE Blood Vessels Sternberg's Diagnostic Surgical Pathology. Volume 1. 5th edition 2009 Philadelphia: Lippincott Williams & Wilkins 1236 1238

[B2] Smetana GW Shmerling RH Does this patient have temporal arteritis? JAMA 2002 287 1 92 101 10.1001/jama.287.1.92 11754714

[B3] Gordon LK Levin LA Visual loss in giant cell arteritis JAMA 1998 280 4 385 386 10.1001/jama.280.4.385 9686559

[B4] Font C Cid MC Coll-Vinent B Lopez-Soto A Grau JM Clinical features in patients with permanent visual loss due to biopsy-proven giant cell arteritis Br J Rheumatol 1997 36 2 251 254 10.1093/rheumatology/36.2.251 9133940

[B5] Liu GT Glaser JS Schatz NJ Smith JL Visual morbidity in giant cell arteritis Clinical characteristics and prognosis for vision. Ophthalmology 1994 101 11 1779 1785 10.1016/s0161-6420(94)31102-x 7800356

[B6] Hellmann DB Temporal arteritis: a cough, toothache, and tongue infarction JAMA 2002 287 22 2996 3000 10.1001/jama.287.22.2996 12052130

[B7] Fule T Mathe M Suba Z Csapo Z Szarvas T Tatrai P Paku S Kovalszky I The presence of human papillomavirus 16 in neural structures and vascular endothelial cells Virology 2006 348 2 289 296 10.1016/j.virol.2005.12.043 16499942

[B8] Gonzalez-Gay MA Vazquez-Rodriguez TR Lopez-Diaz MJ Miranda-Filloy JA Gonzalez-Juanatey C Martin J Llorca J Epidemiology of giant cell arteritis and polymyalgia rheumatica Arthritis Rheum 2009 61 10 1454 1461 10.1002/art.24459 19790127

[B9] Elkind MS Inflammatory markers and stroke Curr Cardiol Rep 2009 11 1 12 20 10.1007/s11886-009-0003-2 19091170

[B10] Ma Z Liu L Zhang F Yu M Wang K Luo J Liu K Chen B Xu L Human papillomavirus type 16 exists in bacteria isolated from cervical cancer biopsies J Int Med Res 2009 37 4 1065 1074 19761689 10.1177/147323000903700411

[B11] Hunder GG Bloch DA Michel BA Stevens MB Arend WP Calabrese LH Edworthy SM Fauci AS Leavitt RY Lie JT The American College of Rheumatology 1990 criteria for the classification of giant cell arteritis Arthritis Rheum 1990 33 8 1122 1128 2202311 10.1002/art.1780330810

[B12] Third Wave Technologies, The Cervista TM HPV HR http://www.cervistahpv.com/pdf/Cervista_HPV_HR_PI_15-3100_Rev_C.pdf

[B13] Weyand CM Schonberger J Oppitz U Hunder NN Hicok KC Goronzy JJ Distinct vascular lesions in giant cell arteritis share identical T cell clonotypes J Exp Med 1994 179 3 951 960 10.1084/jem.179.3.951 8113687 PMC2191412

[B14] Cooper RJ D'Arcy S Kirby M Al-Buhtori M Rahman MJ Proctor L Bonshek RE Infection and temporal arteritis: a PCR-based study to detect pathogens in temporal artery biopsy specimens J Med Virol 2008 80 3 501 505 10.1002/jmv.21092 18205226

[B15] Wagner AD Gerard HC Fresemann T Schmidt WA Gromnica-Ihle E Hudson AP Zeidler H Detection of Chlamydia pneumoniae in giant cell vasculitis and correlation with the topographic arrangement of tissue-infiltrating dendritic cells Arthritis Rheum 2000 43 7 1543 1551 10.1002/1529-0131(200007)43:7<1543::AID-ANR19>3.0.CO;2-8 10902759

[B16] Elling P Olsson AT Elling H Synchronous variations of the incidence of temporal arteritis and polymyalgia rheumatica in different regions of Denmark; association with epidemics of Mycoplasma pneumoniae infection J Rheumatol 1996 23 1 112 119 8838518

[B17] Powers JF Bedri S Hussein S Salomon RN Tischler AS High prevalence of herpes simplex virus DNA in temporal arteritis biopsy specimens Am J Clin Pathol 2005 123 2 261 264 10.1309/2996TT2CTLTKN0KT 15842052

[B18] Cankovic M Zarbo RJ Failure to detect human herpes simplex virus, cytomegalovirus, and Epstein-Barr virus viral genomes in giant cell arteritis biopsy specimens by real-time quantitative polymerase chain reaction Cardiovasc Pathol 2006 15 5 280 286 10.1016/j.carpath.2006.05.007 16979035

[B19] Rodriguez-Pla A Bosch-Gil JA Echevarria-Mayo JE Rossello-Urgell J Solans-Laque R Huguet-Redecilla P Stone JH Vilardell-Tarres M No detection of parvovirus B19 or herpesvirus DNA in giant cell arteritis J Clin Virol 2004 31 1 11 15 10.1016/j.jcv.2004.05.003 15288607

[B20] Helweg-Larsen J Tarp B Obel N Baslund B No evidence of parvovirus B19, Chlamydia pneumoniae or human herpes virus infection in temporal artery biopsies in patients with giant cell arteritis Rheumatology (Oxford) 2002 41 4 445 449 10.1093/rheumatology/41.4.445 11961176

[B21] Njau F Ness T Wittkop U Pancratz T Eickhoff M Hudson AP Haller H Wagner AD No correlation between giant cell arteritis and Chlamydia pneumoniae infection: investigation of 189 patients by standard and improved PCR methods J Clin Microbiol 2009 47 6 1899 1901 10.1128/JCM.02438-08 19386842 PMC2691119

[B22] Haugeberg G Bie R Nordbo SA Chlamydia pneumoniae not detected in temporal artery biopsies from patients with temporal arteritis Scand J Rheumatol 2000 29 2 127 128 10.1080/030097400750001941 10777127

[B23] Nordborg C Nordborg E Petursdottir V LaGuardia J Mahalingam R Wellish M Gilden DH Search for varicella zoster virus in giant cell arteritis Ann Neurol 1998 44 3 413 414 10.1002/ana.410440323 9749614

[B24] Cuschieri KS Cubie HA Whitley MW Gilkison G Arends MJ Graham C McGoogan E Persistent high risk HPV infection associated with development of cervical neoplasia in a prospective population study J Clin Pathol 2005 58 9 946 950 10.1136/jcp.2004.022863 16126875 PMC1770812

[B25] Zur Hausen H Papillomaviruses and cancer: from basic studies to clinical application Nat Rev Cancer 2002 2 5 342 350 10.1038/nrc798 12044010

[B26] Gillison ML Human papillomavirus-associated head and neck cancer is a distinct epidemiologic, clinical, and molecular entity Semin Oncol 2004 31 6 744 754 10.1053/j.seminoncol.2004.09.011 15599852

[B27] Masini C Fuchs PG Gabrielli F Stark S Sera F Ploner M Melchi CF Primavera G Pirchio G Picconi O Petasecca P Cattaruzza MS Pfister HJ Abeni D Evidence for the association of human papillomavirus infection and cutaneous squamous cell carcinoma in immunocompetent individuals Arch Dermatol 2003 139 7 890 894 10.1001/archderm.139.7.890 12873884

[B28] Gnanamony M Peedicayil A Subhashini J Ram TS Rajasekar A Gravitt P Abraham P Detection and quantitation of HPV 16 and 18 in plasma of Indian women with cervical cancer Gynecol Oncol 2010 116 3 447 451 10.1016/j.ygyno.2009.10.081 19922992

[B29] Ho CM Yang SS Chien TY Huang SH Jeng CJ Chang SF Detection and quantitation of human papillomavirus type 16, 18 and 52 DNA in the peripheral blood of cervical cancer patients Gynecol Oncol 2005 99 3 615 621 10.1016/j.ygyno.2005.07.004 16099020

[B30] Wei YC Chou YS Chu TY Detection and typing of minimal human papillomavirus DNA in plasma Int J Gynaecol Obstet 2007 96 2 112 116 10.1016/j.ijgo.2006.08.012 17250836

[B31] Dong SM Pai SI Rha SH Hildesheim A Kurman RJ Schwartz PE Mortel R McGowan L Greenberg MD Barnes WA Sidransky D Detection and quantitation of human papillomavirus DNA in the plasma of patients with cervical carcinoma Cancer Epidemiol Biomarkers Prev 2002 11 1 3 6 11815394

[B32] Yang HJ Liu VW Tsang PC Yip AM Tam KF Wong LC Ng TY Ngan HY Quantification of human papillomavirus DNA in the plasma of patients with cervical cancer Int J Gynecol Cancer 2004 14 5 903 910 10.1111/j.1048-891X.2004.014528.x 15361202

[B33] Brack A Martinez-Taboada V Stanson A Goronzy JJ Weyand CM Disease pattern in cranial and large-vessel giant cell arteritis Arthritis Rheum 1999 42 2 311 317 10.1002/1529-0131(199902)42:2<311::AID-ANR14>3.0.CO;2-F 10025926

